# Next-generation high-throughput in vitro exposure system for early hazard ranking and comparative assessment of cigarette smoke and heated tobacco aerosols

**DOI:** 10.1186/s12302-025-01258-8

**Published:** 2025-11-18

**Authors:** A. Zimmermann-Klemd, L. Wende Wolf, FS. Emser, J. Daniel, M. Follo, R. Trittler, B. Rothen-Rutishauser, P. Deibert, C. Gründemann, M. Garcia-Käufer

**Affiliations:** 1 Translational Complementary Medicine, Department of Pharmaceutical Sciences, University of Basel, Mattenstraße 22, 4058 Basel, Switzerland; 2 Institute for Environmental Research (IFER), RWTH Aachen University, Worringerweg 1, 52074 Aachen, Germany; 3 Lighthouse Core Facility, Department of Medicine I, Medical Center - University of Freiburg, Faculty of Medicine, University of Freiburg, Germany, Hugstetter Strasse 55, 79106 Freiburg, Germany; 4 Department of Pharmacy, Medical Center—University of Freiburg, Faculty of Medicine, University of Freiburg, Germany, Hugstetter Strasse 55, 79106 Freiburg, Germany; 5 BioNanomaterials, Adolphe Merkle Institute, University Fribourg, Chemin Des Verdiers 4, 1700 Fribourg, Switzerland; 6 Institute for Exercise- and Occupational Medicine, Medical Center—University of Freiburg, Faculty of Medicine, University of Freiburg, Germany, Hugstetter Strasse 55, 79106 Freiburg, Germany

**Keywords:** Cigarette smoke, Heated tobacco products, Inhalation hazard assessment, Genotoxicity, Cytotoxicity, Immunomodulation, Air–Liquid Interface (ALI), Continuous-flow exposure, High-throughput screening, 96-well format

## Abstract

**Background:**

Accuracy, along with reproducibility and transferability, are essential attributes of modern in vitro inhalation exposure systems to reliably assess the hazard potential of complex aerosols and remain aligned with advancements in molecular and cellular biology. Although in vitro lung exposure models are continually being refined, no standardized or harmonized methodologies have yet been established or universally accepted. This study addresses the existing gap by evaluating a novel exposure system designed to generate physiologically relevant in vitro inhalation data. The system is based on a high-throughput 96-well platform operating under continuous-flow air–liquid interface conditions, enabling controlled, reproducible, and scalable aerosol exposures. To evaluate exposure system performance, reference cigarette smoke (1R6F) was applied and subsequently compared with heated tobacco aerosol (IQOS) using an alveolar epithelial cell model (A549). The study aimed to demonstrate the reliability of the high-throughput exposure technology and its applicability for emission prioritization using a tiered in vitro test battery.

**Results:**

The high-throughput exposure system (HTES) delivers aerosols to cell based test systems cultured at the air–liquid interface (ALI) in 96-well insert microplates, facilitating the acquisition of consistent and interpretable dose–response relationships. It provides 11 aerosol concentration levels in a single batch, including a vehicle control and eight technical replicates per level, yielding 96 data points. The consistency between nominal dose metrics and induced biological responses, evaluated through a predictive assay panel (cytotoxicity, genotoxicity, immunomodulation), confirms both accuracy and reproducibility. A comparative evaluation of 1R6F and IQOS emissions, supported by chemical analyses, demonstrated the method’s applicability across aerosol types and enabled the determination of relative potency equivalents, which facilitate the ranking of tobacco products. 1R6F smoke exhibited a substantially higher acute potency than IQOS aerosol, although both caused genotoxic effects. Additionally, the successful implementation of high-content assays, such as in situ γ-H2AX analysis, highlights the system’s potential for applications beyond traditional hazard screening.

**Conclusions:**

By combining precise conditioning and uniform dosing of native aerosols with notably low background noise, the HTES enables the generation of valuable dose–response data. The results indicate a close approximation to physiological lung conditions—an outcome that is not inherently expected from a continuous-flow exposure system operating in a novel microplate format.

## Background

Replicating lung physiology while maintaining the native properties of complex inhalable aerosols is crucial for generating reproducible and reliable in vitro assessments of inhalation hazards and drug efficacy. Effect-based data generated from such models can serve as a first-line screening tool, supporting early product development, regulatory submissions, or even health claim substantiation. However, this relies on the ability to verify the physiological relevance and predictive capacity of the inhalation model and in vitro exposure system used in practical applications [1–4].

Instrument specifications for exposing cell-based test systems at the ALI only illustrate a system’s technical potential. Whether these technology is truly suitable for advanced aerosol testing becomes evident only through practical, real-world application [5]. While the rapid generation of comparative data for inhalation hazard is a major strength of in vitro testing, it also highlights limitations in relevance and extrapolation of findings. To date, in vitro-based inhalation assessments remain largely indicative tools, primarily employed for screening purposes [6, 7].

The credibility and acceptance of in vitro-derived health hazard rankings ultimately depend on methodo-logical consistency, transparency, and standardization. Enhancing the human relevance of such systems requires both technological innovation and rigorous, evidence-based validation [8]. These demands place high performance expectations on ALI exposure systems used in conjunction with cell-based lung models [9].

In this context, tobacco product emissions serve as prototypical references for assessing inhalation health hazards and are central to the development and validation of New Approach Methodologies (NAMs). From a physicochemical perspective, aerosols produced by tobacco products—whether through combustion, vaporization, or volatilization—are complex, dynamic mixtures containing gases, volatile organic compounds, and particulate matter [6]. These constituents undergo various transformations, such as thermochemical degradation and humidification, from the point of synthesis and emission through the respiratory tract [10, 11]. This complexity has likely impeded the development of standardized in vitro exposure methods and dose metrics [12, 13], both of which are essential for building a robust knowledge base on their health impacts, especially considering their ubiquity and socio-economic implications.

This gap underscores the critical need for continued innovation in exposure technologies, accompanied by precise dosimetry, and their systematic integration into regulatory testing frameworks. Even when performance parameters of engineered ALI systems are supported by physicochemical models and data, their true value must be demonstrated through integration into biological hazard assessment workflows [12–15].

A central priority in the development of those new technologies is the accurate simulation of lung micro-environments during the exposure event. Several ALI-specific parameters—such as humidity and flow characteristics, despite their recognized importance—have yet to be formally defined or standardized through technical or procedural guidelines [9, 16, 17]. For example, a recent inter-laboratory study using the VITROCELL® Cloud12 system established a detailed standard operating procedure (SOP) for aerosolized solid particle delivery to transwell inserts, confirming the method’s reproducibility and robustness [18]. Beyond ensuring reliable aerosol deposition and uniformity at the apical surface of the in vitro test system, continuous-flow systems must also manage subtle factors such as the humidity of the vehicle (or “clean air”). The vehicle’s impact serves as a baseline reference and directly reflects the performance of humidification systems, which are vital for maintaining epithelial cell homeostasis and to model realistic exposure scenarios. Instability in this baseline may confound the effects induced by the test aerosol, particularly in sensitive molecular assays or during prolonged exposure [19, 20].

These technical and biological requirements guided the design of the HTES (VITROCELL® 96 Exposure System, VITROCELL Systems GmbH, Waldkirch, Germany). The platform features automated temperature regulation alongside real-time monitoring of flow and humidity, ensuring a stable and reproducible microenvironment suitable for acute exposures and sub-toxic mechanistic studies. Temperature control is achieved via integrated heated water circuits, while the humidification system maintains physiological moisture levels to support epithelial cell integrity throughout the exposure. Automation minimizes variability introduced by manual handling, facilitating technology transfer across laboratories—a major barrier to harmonization.

In addition to its automation features, the HTES is distinguished by its unprecedented throughput enabled by a multiwell microplate format. This innovation aligns with the expanding availability of 96-well high-throughput screening (HTS) insert plates, reflecting a co-evolution of hardware and consumables for efficient, large-scale inhalation screening. Downscaling enables comprehensive dose–response analyses within a single exposure run, facilitating derivation of points of departure (PoDs) for efficient prioritization and categorization of test aerosols. The HTES can generate up to 96 data points per endpoint per exposure, including 11 dilution stages with eight technical replicates plus a vehicle (clean air) control. This level of resource efficiency fills a critical gap in the use of in vitro data for aerosol risk assessment [21].

To demonstrate the system’s versatility, we conducted an exploratory proof-of-concept study comparing emissions from two tobacco products: the combustible reference cigarette 1R6F and IQOS, a heated tobacco product (HTP). HTPs are marketed as lower-risk alternatives to conventional cigarettes, due to their reduced combustion-specific emissions [22]. However, their emission profiles and long-term health effects remain poorly characterized [23, 24].

This comparative hazard assessment study, conducted with emissions from two distinct tobacco products, clearly delineates the HTES’s potential applications beyond generic toxicity screening. Moreover, the study emphasized the critical interplay between process engineering and biological effect assessment, adopting an integrated approach to evaluate the system’s capability in capturing relevant endpoints of effect.

## Methods

### Suppliers

Unless otherwise specified, all chemicals, reagents, and cell culture media were obtained from Sigma-Aldrich GmbH (Taufkirchen, Germany), and all disposable consumables were supplied by Sarstedt AG & Co. KG (Nümbrecht, Germany).

### Tobacco product test items

The combustible reference product used in this study was the 1R6F Reference Cigarette (University of Kentucky), designed to represent commercially available “full-flavored” American-blend filtered cigarettes. The 1R6F is a well-characterized standard widely used in both regulatory and academic research [25]. Cigarettes were conditioned prior to use following ISO 3402 guidelines [26]. As a representative HTP, the IQOS Iluma™ system (Philip Morris International), a commercially available heat-not-burn device using tobacco heat sticks, was selected. Both the 1R6F cigarettes and IQOS tobacco sticks were stored at room temperature in their original sealed packaging. Only freshly opened packages were used for experiments to ensure product integrity and reproducibility.

### Test aerosol generation from 1R6F cigarette smoke and IQOS emissions

Test aerosol generation was carried out using the VITROCELL Smoking Machine VC 1®, in accordance with the ISO 20778:2018 guideline. Although originally designed for conventional cigarette smoke, the VC 1 has been validated for use with alternative tobacco products, including HTPs and e-cigarettes [27, 28]. The smoking machine delivers volumes of test aerosol intermittently to the HTES according to standardized puffing profiles. An intense smoking regimen was applied for both 1R6F reference cigarettes and IQOS heat sticks, following the specifications outlined in the CORESTA Recommended Method No. 81 (CRM81, 2015) and relevant technical guidelines [26, 29, 30]. Each puff had a volume of 55 mL, drawn over 3 s, at 30-s intervals, using a square wave puffing profile. This setup ensures consistency and reproducibility in aerosol generation, thereby enabling meaningful comparisons between combustible tobacco and HTP exposures.

### High-throughput exposure system: aerosol dilution, humidification, and delivery

**Fig. 1:**
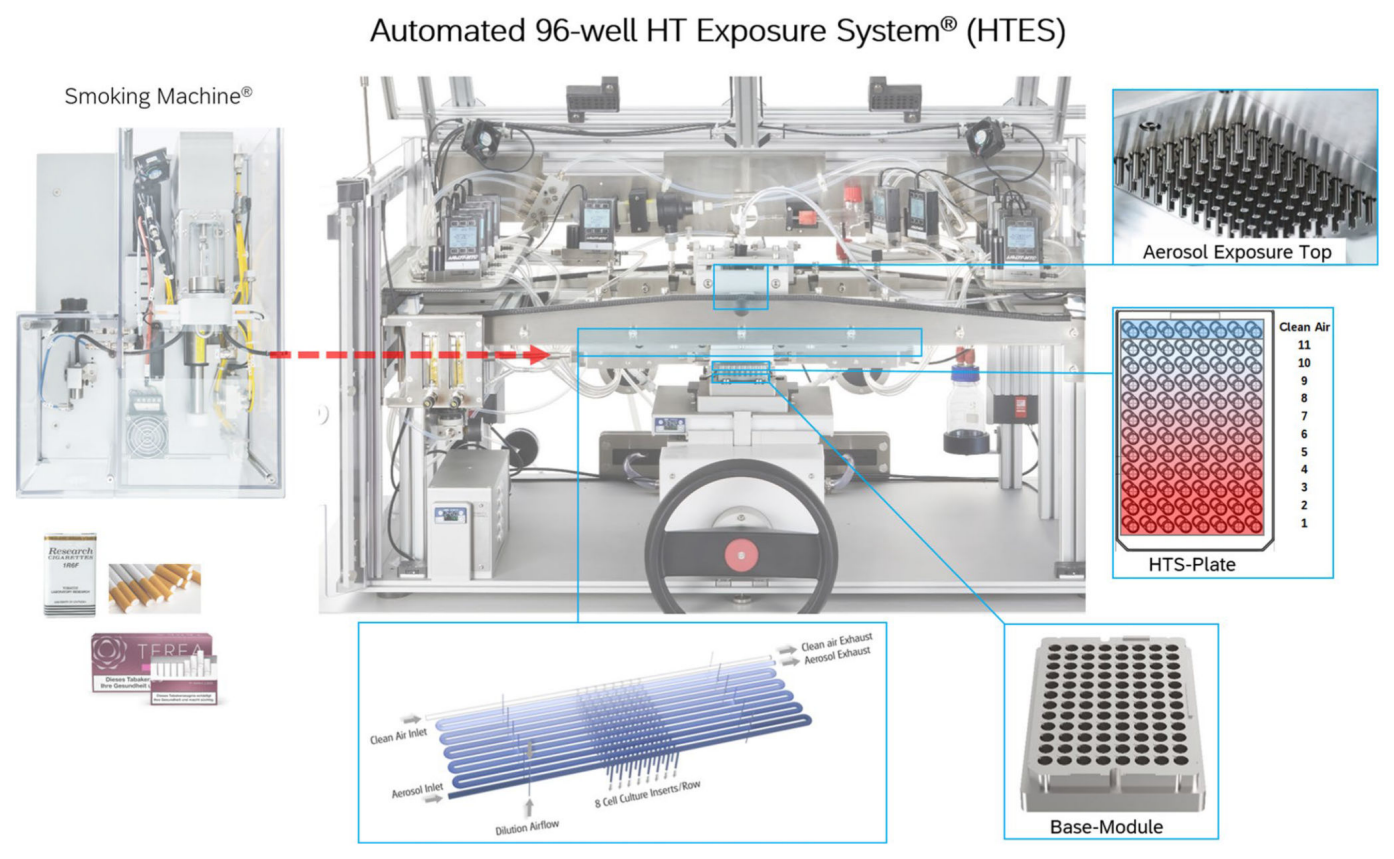
High-throughput 96-well air–liquid interface (ALI) exposure system (HTES) by VITROCELL Systems, featuring exploded views of the top and bottom modules that illustrate the distribution and arrangement of aerosol dilution levels across the HTS microplate. Whole aerosol is generated by the Smoking Machine VC1® (VITROCELL Systems), which is actuated via software control. The aerosol is actively transferred to the HTES through inert Iso-Versinic® tubing (aerosol flow indicated by the red arrow). Upon entering the HTES, the first dilution step is performed based on pre-defined settings. Simultaneously, humidification occurs through mixing with vehicle (clean air), producing the test atmosphere delivered to the first column of the HTS plate (representing the highest aerosol concentration level). A total of eleven distribution nodes enable a sequential dilution cascade, where the main aerosol flow is progressively mixed with humidified clean air. Each dilution stage is precisely controlled by individual mass flow controllers (MFCs), allowing accurate and reproducible aerosol dosing across the microplate

The HTES is a semi-automated, continuous-flow aerosol exposure platform designed to deliver test aerosols to epithelial lung cells cultured in HTS insert plates (Fig. 1). It builds upon the established 24/48-well exposure systems from VITROCELL Systems, preserving their modular architecture while incorporating further miniaturization to meet the demands of high-throughput applications. The system ensures precise control over aerosol flow rates, mixing efficiency and relative humidity of the vehicle, enabling accurate and reproducible dosing.

The HTES is connected to the Smoking Machine VC 1® (VITROCELL Systems), which serves as the aerosol generator for the investigated tobacco products. The whole aerosol generated by the VC 1 is actively transported into the HTES, where controlled turbulent mixing with a humidified vehicle stream (“clean air”) combined with laminar flow regions achieves uniform aerosol dilution, forming the first and highest exposure level. Subsequently, the aerosol undergoes automated, multi-stage sequential dilution to generate 11 precisely defined concentration levels. Dilution is governed by mass flow controllers (MFCs; Analyt-MTC® Messtechnik GmbH, Germany), which deliver defined flows of humidified clean air at designated nodal points upstream of each exposure level. Each nodal point corresponds to a single column of the 96-well HTS plate, with column 12 corresponding to the vehicle or clean air control. Cells in this column are exposed solely to humidified air.

Fractions of test aerosol dilutions are ultimately distributed to 96 individual inlets, delivering a continuous mass flow of 1.2 mL/min per well. This continuous flow, driven by vacuum and regulated through flow restictors, ensures stable and reproducible exposure at the apical surface of ALI-cultured cell monolayers.

Humidification of the vehicle (HEPA-filtered) is achieved using an integrated Humidification Station® (VITROCELL Systems), set to 90 ± 2% relative humidity at 37 °C via the VITROCELL RH/T Controller®. The vehicle control (column 12) enables evaluation of non-specific effects induced by the vehicle alone, when compared to an unexposed incubator control. This setup serves as an indicator of the system’s baseline performance, as well as for quantifying potential background exposure effects.

### Assessment of aerosol deposition efficiency

Accurate aerosol dosimetry—particularly the quantification of particle deposition efficiency and delivery of volatiles at the apical cell surface—is essential for interpreting biological effects in continuous-flow ALI exposure systems. It provides essential data for defining dose–response relationships, facilitating comparison with existing literature, and cautiously extrapolating findings to human exposure scenarios.

The assessment of distribution efficiency and accuracy across the 96 single inserts of the exposure module was adapted to the specific aerosol under investigation. For combustible cigarette smoke (1R6F), deposition of total particulate matter (TPM) was measured using auto-fluorescence emission. A stainless steel 96-well “dummy plate” (VITROCELL Systems), prefilled with 100 μL of DMSO (“trapping solution”) per well, was therefore used as replacement for the HTS cell culture insert plate. Following a simulated in vitro exposure (see Sect. 2.8), 70 μL aliquots of TPM-DMSO solution were transferred to an F-bottom 96-well plate, and autofluorescence was measured (relative fluorescence units, RFU485; Tecan M200).

For the IQOS aerosol, which is rich in glycerol due to its common use as a humectant in HTPs [31], an enzymatic free glycerol reagent (100 μL per well) was used to relatively quantify delivery of aerosol at the site of exposure. The reaction product’s optical density was measured at 540 nm (OD540) from a 70 μL aliquot transferred to an F-bottom plate.

#### Carbonyl compound analysis in test aerosols (HPLC–DAD)

Sampling for semi-quantitative chemical analysis was performed independently of the cell exposure experiments but under otherwise comparable conditions. A total of 880 mL of cigarette smoke (2 × 8 puffs from 2 cigarettes) was drawn through a single impinger containing a 2,4-dinitrophenylhydrazine (DNPH) derivatization solution, following procedures described in ISO 21160:2018 and CORESTA CRM 74 guidelines [32, 33]. The resulting extract, containing aldehyde and ketone hydrazone derivatives, was analyzed by reverse-phase high-performance liquid chromatography (RP-HPLC) and quantified using diode array detection (DAD). DNPH derivatives were identified and quantified based on carbonyl calibration standards, enabling semi-quantitative determination of selected carbonyl compounds in mainstream cigarette smoke. Materials and the analytical method employed were as follows: Column: Luna C18(2) (00G-4252-E0); Dimensions: 250 mm × 4.6 mm ID; Particle Size: 5 μm; Pore Size: 100 Å; Temp.: 30 °C; Inj. Vol.: 10 μL; Solvent A: 30% Acetonitrile, 10% Tetrahydrofuran (THF), 1% Isopropyl alcohol (IPA) in Type I water, filtered and degassed (UHP Helium sparged). Solvent B: 65% Acetonitrile, 1% THF, 1% IPA in Type I water, Solvent C: Acetonitrile (UHP Helium sparged). Detector: DAD @ 365 nm, 4.0 nm; Instrument: Shimadzu LC-20A (RP-HPLC). DNPH derivatives were identified and quantified based on a carbonyl calibration standard (Supelco CRM44933, Aldehyde/Ketone-DNPH Stock Standard-13; 15 μg/mL).

### Cell culture

Human alveolar epithelial cells (A549, ECACC #86012804, certified mycoplasma-free) were cultured in T-75 flasks using Dulbecco’s Modified Eagle Medium (DMEM; Gibco, Thermo Fisher Scientific), supplemented with 2 mM L-glutamine, 1% penicillin/streptomycin, and 10% (v/v) fetal bovine serum (Gibco, Thermo Fisher Scientific). All experiments were conducted using cells between passages 3 and 15. At ~ 80% confluence, cells were detached using trypsin–EDTA and seeded into ThinCert® 96-well HTS insert plates (Greiner Bio-One, #655641) at an initial density of 12,500 cells in 70 μL per insert (equivalent to 1.8 × 10^5^ cells/mL or approximately 900 cells/mm^2^). The corresponding receiver plates were filled with 250 μL of growth medium per well to establish the basolateral compartment.

Following 24 h of submerged culture, ALI conditions were initiated by transitioning to air-lift. Cells were then maintained for an additional 24 h under standard incubator conditions (37 °C, 95% relative humidity, 5% CO_2_), resulting in a morphologically confluent monolayer adapted to ALI conditions and ready for aerosol exposure experiments. The protocol for ALI culture establishment was developed based on internal historical data and corroborated by previous studies conducted at our facility [27].

### Air–liquid interface exposure

Prior to aerosol exposure, cell cultures conditioned at the ALI (see Sect. 2.7) in HTS plates were transferred from the standard receiver plate to a pre-warmed stainless steel receiver plate (VITROCELL Systems), prefilled with 200 μL of exposure medium (serum-free DMEM supplemented with 25 mM HEPES). This setup ensured direct contact between the insert membranes and the basolateral medium.

The assembly containing the HTS insert plate was immediately installed into the core exposure module of the HTES, housed within the pre-heated (37 °C) climate chamber. Inside the module, the cell-containing HTS insert plate was mechanically sealed between the apical and basolateral compartments using full-surface silicone seals, ensuring airtight integration. Upon sealing, a continuous flow of humidified clean air, which also serves as the vehicle control, was initiated seamlessly to allow system equilibration. Following a 5-min equilibration period, A549 cells were exposed to the test aerosols at 11 different concentration levels: either 1R6F cigarette smoke generated from 7 cigarettes, or IQOS aerosol produced from 21 heat sticks. Each cigarette or heat stick delivered 8 puffs of 55 mL each. The total residence time of the cells in the HTES was 38 min for 1R6F, including 28 min of net aerosol exposure and two 5-min clean air equilibration phases (pre- and post-exposure). For IQOS, the net exposure duration was three times longer—1 h and 24 min—due to the higher number of units (heat sticks) administered to achieve the desired effects.

This inherent difference in exposure duration has significant implications for dosimetry. Therefore, a threefold correction factor was applied when calculating threshold toxicity values to account for the differing dosing durations between the two test articles (see Sect. 3.4). Following exposure, the HTS insert plate was returned to its original receiver plate containing fresh, complete culture medium and placed back into the incubator for a 24-h post-exposure period. All steps were performed promptly to minimize potential non-specific effects on cell viability from prolonged ambient humidity exposure..

### Aerosol dosage range determination

This approach established a well-defined concentration range with measurable effects while maximizing the utilization of all 11 dilution levels. Range-finding tests set the upper toxicity threshold—the concentration and exposure time causing a measurable biological effect—for each test article. The highest dose reliably induced significant effects on apical cellular endpoints, benchmarked against a vehicle control. This ensured a dynamic and interpretable concentration–response curve across the dilution range.

#### Cytotoxicity (LDH Assay) and cell viability (WST-1 Assay)

Lactate dehydrogenase (LDH) release into the basolateral supernatants of aerosol-exposed cells was measured as a surrogate marker of acute cytotoxicity, indicating loss of cell membrane integrity. For normalization and as a positive control, complete cell lysis was induced by adding 1% Triton™ X-100 to the apical surface of the cell monolayer, in a volume equal to the basolateral compartment (200 μL). The assay was carried out according to the manufacturer’s instructions (Cytotoxicity Detection Kit, Roche #11644793001). After a 30-min incubation at room temperature, absorbance was measured at 490 nm (Tecan® M200).

Cell viability was assessed using the WST-1 Cell Proliferation Reagent (Roche #11644807001), diluted to 10% (v/v) in DMEM as per the manufacturer’s protocol. A volume of 70 μL of the pre-warmed reagent was added to the apical side of the cell monolayer. Following a 45-min incubation at 37 °C, 50 μL aliquots were transferred to F-bottom microplates for spectrophotometric analysis of the metabolically converted formazan dye.

Since the WST-1 assay inherently affects cellular metabolic activity, only half of one HTS plate column was used for this assay, preserving four technical replicates per exposure level for subsequent downstream analyses (see Sect. 2.11).

### DNA damage assessment (comet assay)

The alkaline comet assay was performed as described by [34] to detect both single- and double-stranded DNA breaks. Each experiment included a clean air control—corresponding to column 12 on the exposure plate—and an untreated incubator control. As a positive control, cells were exposed to ethyl methanesulfonate (1.25 mM) from the basolateral side for 24 h. The experimental workflow was as follows: Cells were detached from the inserts via trypsinization, pooled from four technical replicates, and 70,000 cells were embedded in low-melting-point agarose on pre-coated microscope slides (prepared in duplicate). Following cell lysis (1 h), DNA unwinding (30 min), and electrophoresis (25 V, 300 mA, 30 min), slides were neutralized using phosphate-buffered saline (PBS) and double-distilled water, then dehydrated with absolute ethanol. Prepared slides were stored at 4 °C until analysis.

For visualization, slides were stained with 70 μL ethidium bromide (10 μM in PBS) and scored by fluorescence microscopy (Leica DMLS) equipped with a CMOS camera. At least 300 comets (nucleoids) were randomly evaluated per technical replicate.

DNA damage was quantified as the percentage of DNA in the comet tail (% DNA in tail) using OpenComet®, an open-source software enabling semi-automated image analysis [35]. It should be noted that due to the series of intricate manual steps required, this assay is not yet compatible with high-throughput screening formats.

### H2AX phosphorylation (γ-H2AX assay)

Phosphorylation of the histone variant H2AX at serine 139, resulting in the formation of γ-H2AX foci, is a rapid and sensitive cellular response to DNA double-strand breaks (DSBs). This modification plays a critical role in the detection and repair of DSBs and has been implicated as a key marker of cellular senescence [36]. Traditionally, γ-H2AX is quantified by manually counting individual nuclear foci. In this study, we employed a more scalable approach by combining fluorescence immunostaining with high-content microscopy, enhanced by artificial intelligence and deep-learning algorithms for quantitative image analysis.

Following aerosol exposure, cells cultured in HTS microplates were processed in situ for immunofluo-rescence microscopy. The protocol began with fixation using 4% paraformaldehyde (PFA), followed by blocking with bovine serum albumin (BSA) and incubation with primary and secondary antibodies, as per the manufacturer’s instructions (HCS DNA Damage Kit, Cat. No. H10292, Invitrogen Ltd.).

Microscopic imaging was performed using a semi-automated high-content screening platform (Olympus scanR), equipped with a 20x/NA 0.45 LUCPLFLN long working-distance objective. Nine fields per well were captured using Hoechst 33,342 for nuclear staining and Cy3 for γ-H2AX signal detection.

Quantitative image analysis was conducted using scanR software. A predefined fluorescence threshold for γ-H2AX intensity was applied to identify nuclei exhibiting DNA damage-associated phosphorylation. The number of positively stained nuclei was normalized to the total number of Hoechst-stained nuclei, enabling semi-quantitative assessment of γ-H2AX induction and, by extension, relative DNA destabilization activity.

### Immunomodulatory response (multiplex ELISA)

Cell culture supernatants were collected from the basolateral compartment of exposed and post-incubated cells and stored at –80 °C until further analysis. Targeted analytes included Interleukin-8 (IL-8), Monocyte Chemoattractant Protein-1 (MCP-1), Epithelial Neutrophil-Activating Protein-78 (ENA-78), and Growth-Regulated Protein Alpha (GROα).

Multiplex cytokine profiling was performed using the LEGENDplex™ assay system (BioLegend Inc.), following the manufacturer’s protocol. Data acquisition was carried out via flow cytometry (Gallios™, Beckman Coulter GmbH), and analyte quantification was completed using the LEGENDplex™ Data Analysis Software.

To validate the responsiveness of the A549 alveolar epithelial test system, known inducers of inflammation were used as positive controls: Tumor Necrosis Factor-alpha (TNFα) and Interleukin-1 beta (IL-1β), each at 6.25 ng/mL, and Lipopolysaccharide (LPS) at 1.25 μg/mL. These controls confirmed the system’s capacity to elicit and sustain pro-inflammatory cytokine responses.

### Statistical analysis and dose–response modelling

Unless otherwise stated, all cellular endpoints were assessed in at least three independent biological replicates (N = 3), with a minimum of two technical replicates per experiment. Statistical analyses were performed using GraphPad PRISM™ (v9.3.1, GraphPad Software). Data normality was evaluated using the Shapiro–Wilk and Kolmogorov–Smirnov tests. Based on the distribution, either parametric (ANOVA) or non-parametric (e.g., Kruskal–Wallis) tests were applied, followed by appropriate multiple comparison procedures (e.g., Holm-Sidak, Dunnett’s, or Dunn’s post hoc test).

For ALI experiments, toxicity data from continuous-flow exposed cells (test aerosols and vehicle controls) were compared to untreated incubator controls. This comparison served to detect any non-specific effects associated with the vehicle (clean air), which may indicate technical deficiencies in maintaining physiological ALI conditions. Statistical significance in comparison to control values was defined as p < 0.05.

Dose–response relationships were modeled and evaluated using the U.S. Environmental Protection Agency’s Benchmark Dose Software (BMDS) version 3.3.2, which provides access to multiple dose–response models and recommends the best-fitting model based on statistical criteria. The benchmark response (BMR) is defined as a predetermined degree of change in a biological end-point relative to the control group, which in this case is set as a 10% change from the baseline value. It represents the response level at which the benchmark dose (BMD) and its lower confidence limit (BMDL) are estimated, providing a statistically based PoD that supports early-stage prioritization in hazard assessment. The Hill model was applied and the PoDs were calculated for early cellular responses, including cytotoxicity, genotoxicity, and immunomodulatory effects, relative to vehicle control [37–39].

The response factors for each toxicity endpoint was calculated between 1R6F and IQOS emissions to compare the inhibitory potency between the two test items. 1R6F served as the reference and was set to 100%. The response factor (RF) was calculated using the formula: RF(%) = (BMDL_1R6F_/BMDL_IQOS_) × 100. This calculation normalizes IQOS potency relative to 1R6F facilitating direct comparison and relative assessment.

## Results

### Aerosol distribution uniformity and deposition efficiency

The exposure system is technically designed to ensure complete and homogeneous mixing of the test aerosol within the main flow conduits, achieved by mass flow controller (MFC)-regulated injection of clean air at specified dilution levels (Fig. 1). Due to the inherent physicochemical variability and behavior of whole aerosols, it is essential to empirically verify the uniformity of aerosol distribution and deposition across the HTS insert plate rather than relying solely on theoretical predictions.

To this end, cell-free exposure simulations (see Sect. 2.5) were conducted. The pre-set volumetric flow rates of the test aerosol were then compared with empirical deposition measurements to validate aerosol delivery accuracy. TPM levels were quantified for 1R6F cigarette smoke, and glycerol concentrations were used as a marker for IQOS aerosol deposition.

**Fig. 2:**
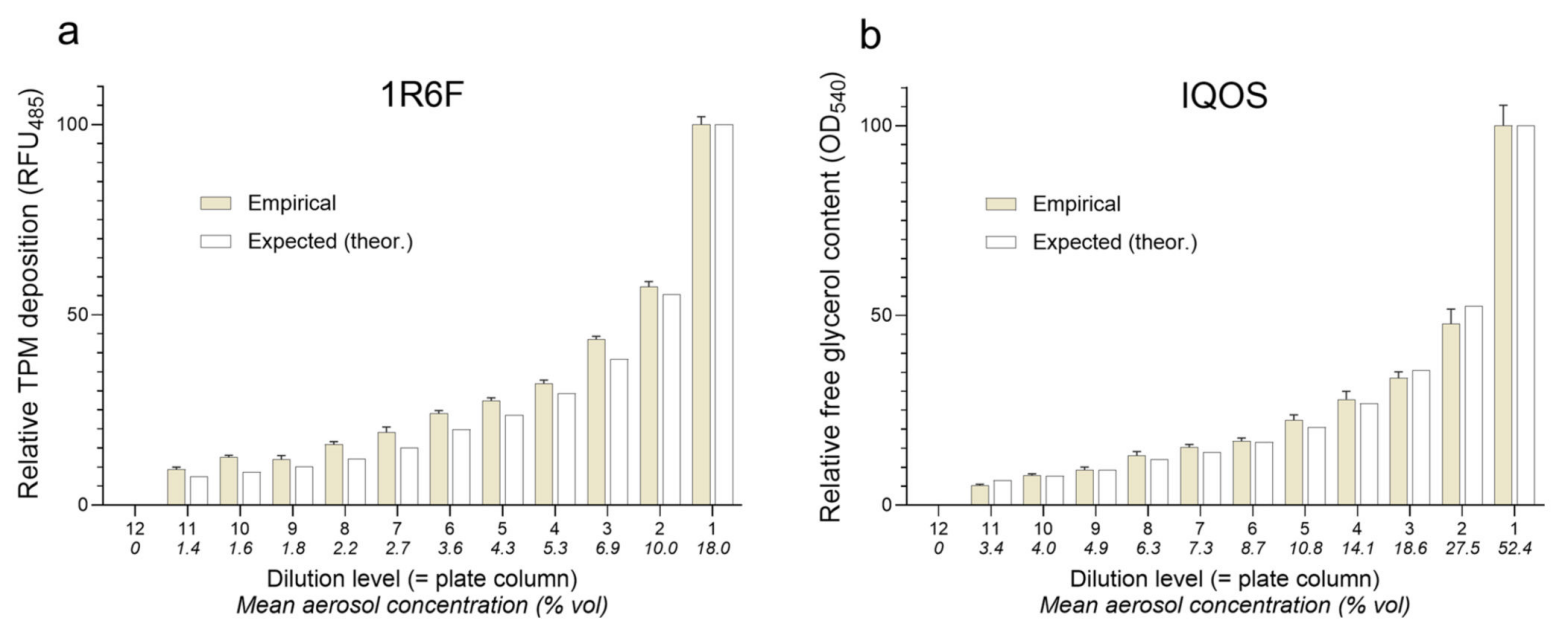
Comparison of expected aerosol concentrations with empirical deposition data in the HTES exposure module: **a** TPM deposition from 1R6F cigarette smoke and **b** glycerol-based quantification from IQOS emissions. Each data point represents the mean of eight technical replicates across three independent experiments (Mean ± SD; N = 3). Values are normalized to 100% (mean deposition at the lowest dilution level) and 0% (blank value from the clean air control)

For both test items, the measured deposition data showed a strong correlation with the theoretical mass flow-based values. Minimal variation was observed among replicates within each dose level (i.e., HTS-plate column), indicating consistent and homogeneous aerosol distribution across the wells (Fig. 2a, b). These results demonstrate that the system consistently delivers reproducible aerosol deposition patterns. Moreover, the mass flow and dilution settings—determined with two distinct aerosol types—can be reliably used to establish dose–response relationships.

### Carbonyl compounds in test aerosols

**Fig. 3:**
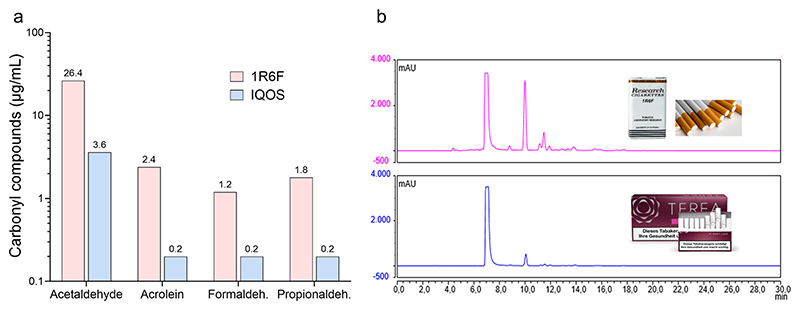
Quantification of the four major aldehydes in test aerosols from 1R6F reference cigarette smoke and the IQOS heated tobacco product using HPLC-DAD. **a** Bars indicate the relative amounts of carbonyl compounds per puff (55 mL), captured in DNPH impinger solution. **b** Representative chromatograms of 1R6F (red trace) and IQOS (blue trace) aerosol samples

The identified compounds, ranked in descending order of concentration, were acetaldehyde, acrolein, propionaldehyde, and formaldehyde. Semi-quantitative analysis revealed that the concentrations of these compounds were up to 20 times higher in 1R6F cigarette smoke compared to IQOS aerosol, with variations depending on the specific compound (Fig. 3). These results highlight the substantial chemical composition differences between emissions from combustible cigarettes and heated tobacco products.

### Preliminary dose range-finding

Following confirmation of aerosol dosing parameters (Sect. 3.1), cell-based experiments using the HTES began with range-finding studies for both aerosol samples (1R6F and IQOS). These studies utilized the full dilution range available on the 96-well HTS plate. The upper dose limit was established based on acute cytotoxicity results from WST-1 and LDH assays, identifying exposure levels that induced statistically significant effects. Table 1 summarizes the established dilution levels, including the corresponding mean and peak aerosol concentrations as well as the spacing factors across plate columns 1 to 11. The determined effective nominal aerosol concentrations and exposure durations differed substantially between 1R6F cigarette smoke and IQOS aerosol.

### Cytotoxicity and cell viability

**Fig. 4:**
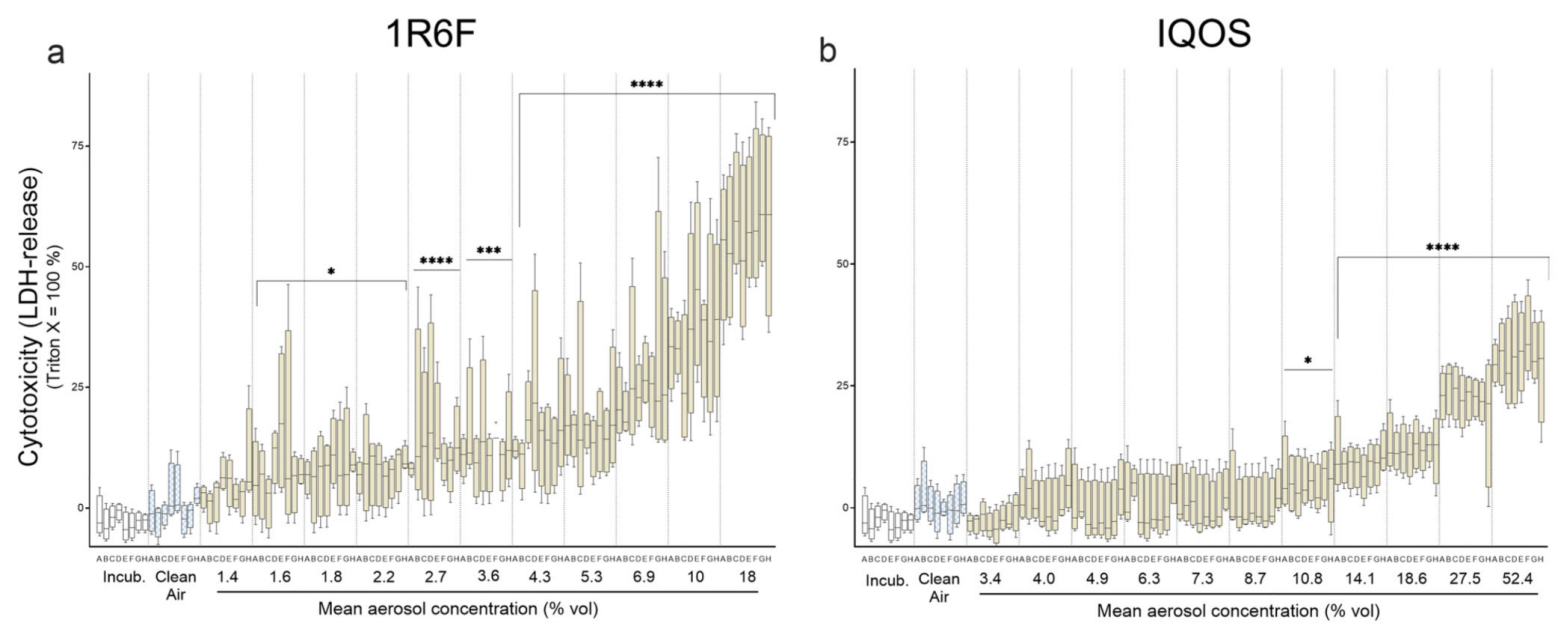
Cytotoxicity assessment (LDH release) in A549 cells following exposure to **a** 1R6F cigarette smoke and **b** IQOS aerosol. Data were normalized to Triton X-100 treatment (100%) and the clean air control (0%). Each boxplot represents data distribution from four independent experiments. Letters A–H on the x-axis denote row positions within the HTS plate. Statistical analysis: two-way ANOVA with Dunnett’s post hoc test (Mean ± SD, N = 4)

**Fig. 5:**
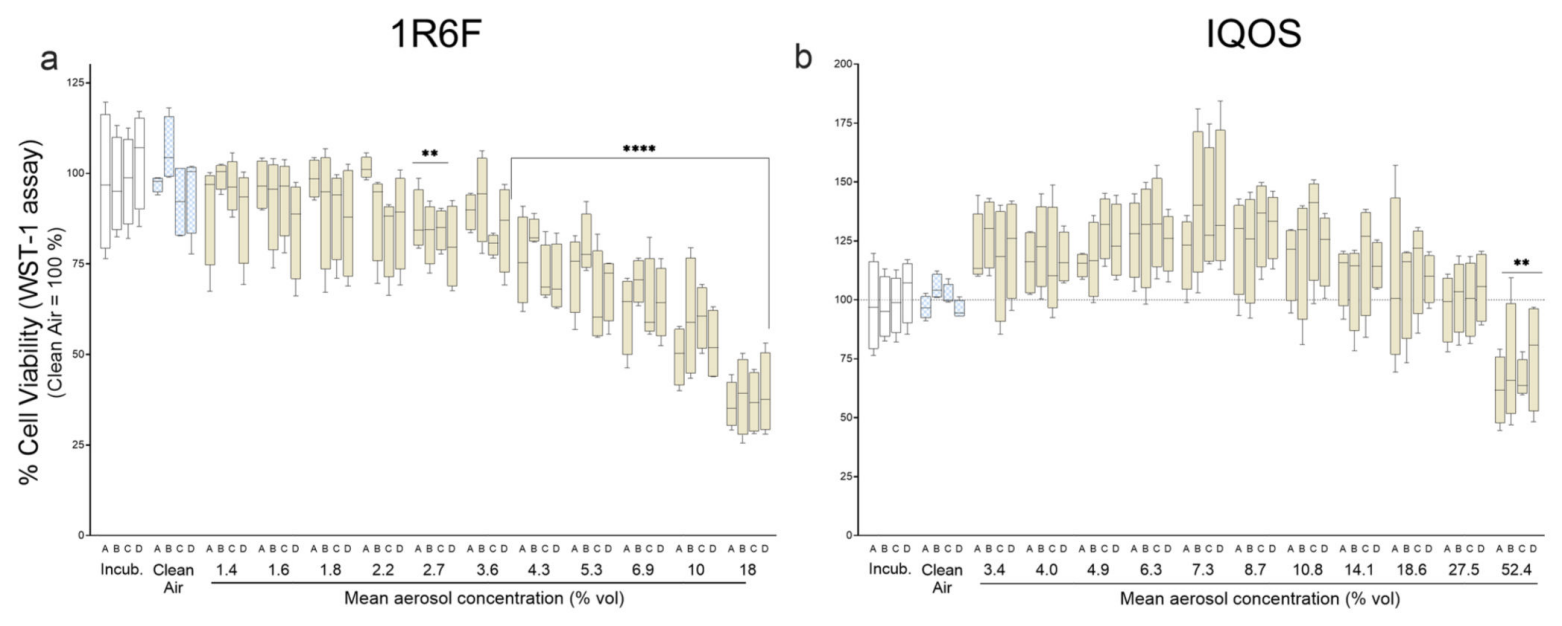
Effect on cell viability (WST-1 assay) in A549 cells after exposure to **a** 1R6F and **b** IQOS aerosol. Results were normalized to the mean value of the clean air control (100%). Letters A–D on the x-axis indicate row positions within the HTS plate. Statistical analysis: two-way ANOVA with Dunnett’s post hoc test (Mean ± SD, N = 4)

Cytotoxicity and cell viability following exposure to 1R6F and IQOS aerosols were assessed in A549 cells using complementary assays. A concentration-dependent increase in LDH release (indicative of cell membrane damage) and a corresponding decrease in metabolic activity (WST-1 assay) were observed for both aerosols (Figs. 4and 5). However, 1R6F smoke exhibited a markedly higher toxicity compared to IQOS aerosol.

Boxplots illustrate variability within technical replicates per column—eight for LDH and four for WST-1—providing a visual assessment of reproducibility. Importantly, IQOS required substantially higher aerosol doses to achieve comparable effects. For instance, an IC_50_ response was reached with 56 puffs from seven 1R6F cigarettes (28 min exposure), whereas 168 puffs from 21 IQOS sticks (84 min exposure) were needed to induce similar effects. The peak aerosol concentration tested for IQOS was approximately threefold higher than for 1R6F (52.4% vs. 18% v/v).

LDH release showed a clear dose–response relationship for both aerosol samples (Fig. 4a, b). In contrast, the WST-1 response to IQOS aerosol exposure exhibited a non-monotonic pattern (Fig. 5b), making it unsuitable for PoD estimation, as reliable PoDs require a consistent and predictable dose–response relationship. The increased exposure duration in IQOS experiments was factored in as a multiplier when calculating the BMDL. However, due to the nonlinear and chemical-specific relationship between exposure duration and toxicity, simple scaling of PoD values by exposure duration may not accurately reflect the relative potency of IQOS emissions. Therefore, adjustments to account for differences in exposure duration should be interpreted with caution, and further modeling of concentration–duration-response relationships would strengthen the comparisons. Overall, the acute cytotoxic potential of IQOS was significantly lower than that of 1R6F, as reflected by the endpoint-specific BMDL values across all assessed endpoints (Table 2).

### DNA damage

**Fig. 6:**
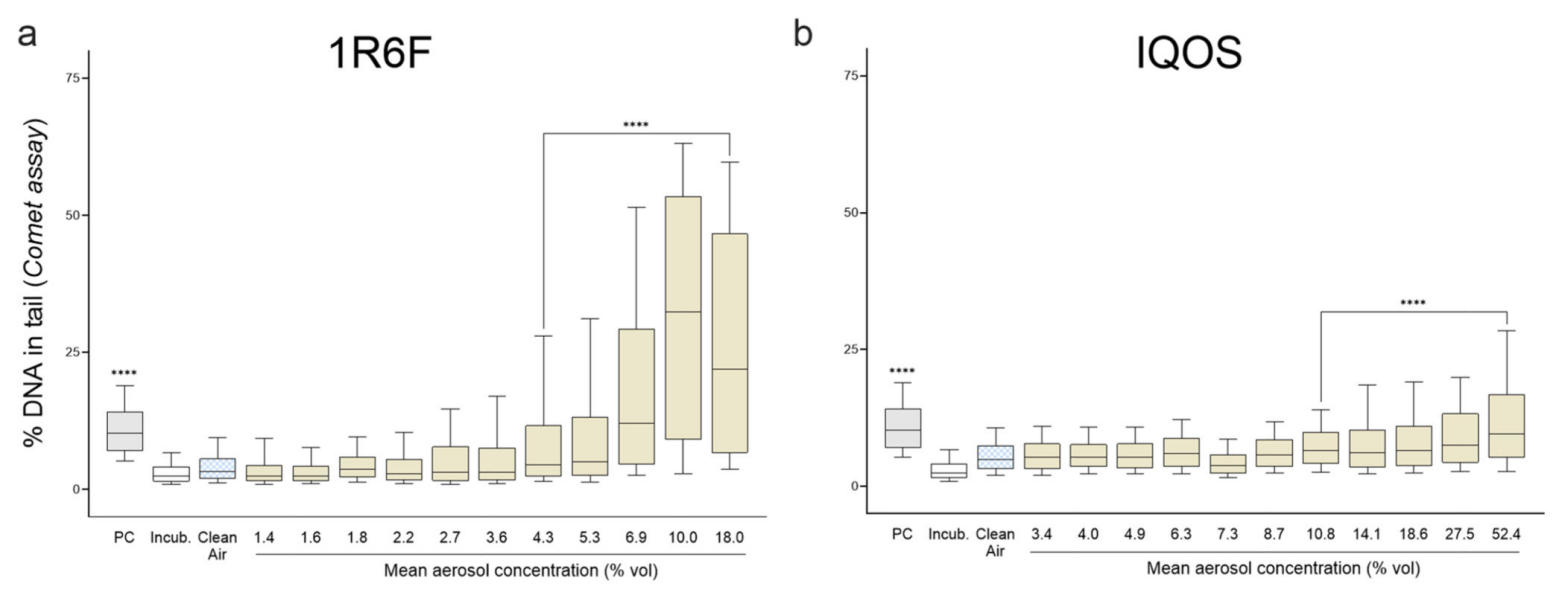
DNA strand break induction in A549 cells exposed to **a** 1R6F cigarette smoke and **b** IQOS aerosol, as measured by the comet assay (% DNA in tail). Statistical analysis: one-way ANOVA followed by Kruskal–Wallis test (N = 3)

The alkaline comet assay demonstrated a dose-dependent induction of both single- and double-stranded DNA breaks in A549 cells exposed to 1R6F cigarette smoke and IQOS aerosols. Although the concentration of IQOS exposure was sixfold higher—due to a lower dilution factor and extended exposure duration—its genotoxic potency remained significantly lower. BMDL comparisons indicate that IQOS aerosol exhibits only approximately 5% of the genotoxic potency of 1R6F (Fig. 6b; Table 2).

### γ-H2AX

**Fig. 7:**
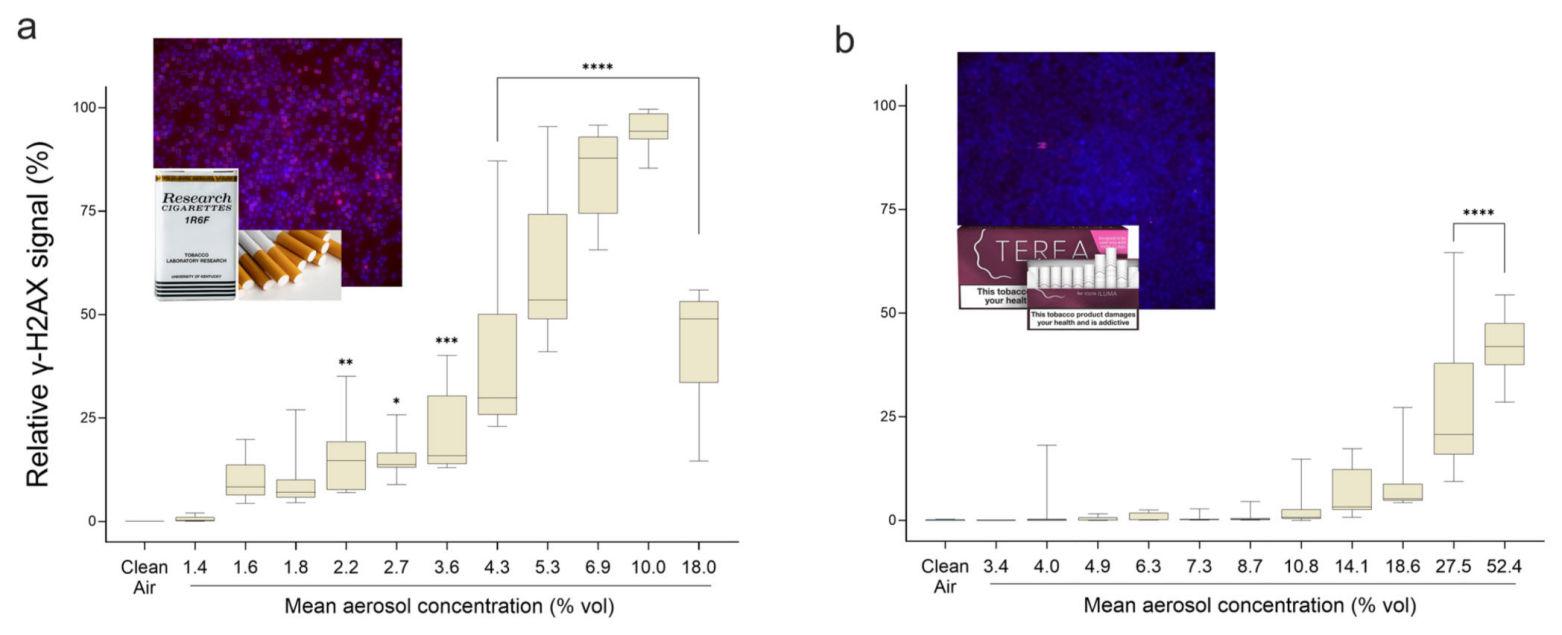
γ-H2AX induction in A549 cells following exposure to **a** 1R6F and **b** IQOS aerosol, assessed by fluorescence microscopy and cytometric analysis. The boxplots display the median values and variability derived from eight technical replicates. Inset immunofluorescence images illustrate the differential genotoxic potential of 1R6F (5.3% vol) and IQOS (6.3% vol); red foci indicate γ-H2AX-positive nuclei, while blue staining (Hoechst 33,342) marks the nuclear background

Phosphorylation of histone H2AX (γ-H2AX) was used as a complementary and sensitive marker of induced DNA damage and genomic instability. 1R6F smoke induced γ-H2AX foci formation starting at an aerosol concentration of 2.2% vol. (Fig. 7a, b). In contrast, IQOS aerosol required a substantially higher concentration (27.5% vol.) to elicit a statistically significant response, even after accounting for the extended exposure duration (p < 0.05).

### Immunomodulatory response

**Fig. 8:**
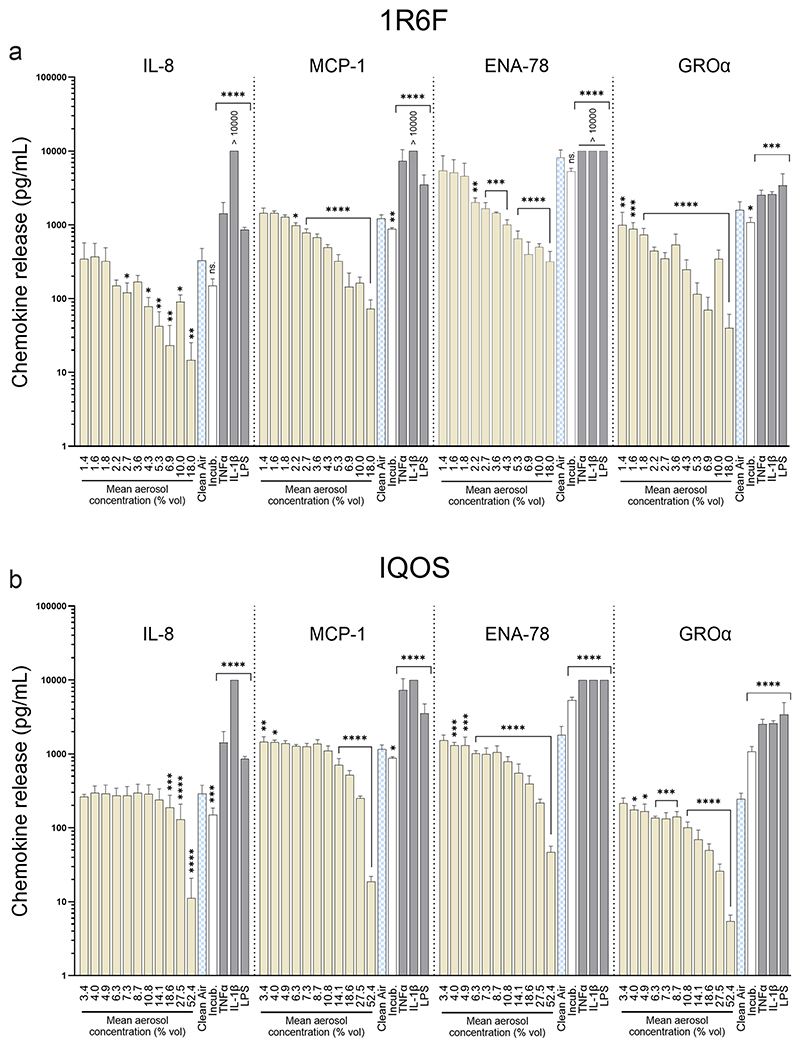
Chemokine release into the basolateral supernatant of A549 cells exposed at the ALI to **a** 1R6F cigarette smoke or **b** IQOS aerosol. TNFα/ IL-1β (12.5 ng/mL) and LPS (2.5 μg/mL) served as positive controls. Results are shown as mean ± SD (N = 3). Statistical analysis: two-way ANOVA with Dunnett’s post hoc test

Selected chemokines—IL-8 (CXCL8), MCP-1 (CCL2), ENA-78 (CXCL5), and GROα (CXCL1)—were analyzed for their roles in modulating alveolar epithelial inflammatory responses. Positive controls (LPS, TNFα, IL-1β) produced a consistent upregulation of all target analytes (Fig. 8a, b), confirming assay sensitivity. In aerosol-exposed cells, 1R6F smoke induced a pronounced immunomodulatory effect, even at sub-cytotoxic doses, while IQOS aerosol showed a substantially weaker response.

Overall, cytokine secretion followed a trend of dose-dependent decrease, consistent with an immunosuppressive effect. However, further decreases observed at the highest (cytotoxic) exposure levels should be interpreted with caution, as they may reflect secondary effects due to cytotoxicity rather than specific immunomodulation. Multiplex cytokine profiling of basolateral supernatants further demonstrated the HTES system’s capacity for high-resolution mechanistic analysis.

## Discussion

### Addressing challenges in air–liquid interface exposure using advanced high-throughput technologies

The development of the 96-well HTES, through strong interdisciplinary collaboration between engineering and life sciences, was designed to address known shortcomings in ALI exposure methodologies, particularly under continuous-flow exposure conditions, by ensuring stable, reproducible aerosol delivery and improved cell viability.

A central goal was to create a platform with standardization potential, which is essential for generating reproducible, high-quality toxicity data that approximate human-relevant exposures [6]. Next-generation NAMs should be modular, scalable, and technically transferable to facilitate regulatory adoption while adhering to the 3R principles. The internal validation procedure highlighted several key outcomes:

#### (a) Increased throughput while preserving precise aerosol distribution

Empirical dose–response patterns aligned closely with theoretical aerosol deposition values, confirming consistency and internally validating the exposure system (Fig. 2). The microplate format preserves or even enhances physiological relevance, while automation improves reliability and reproducibility. Importantly, the system’s modular design supports applications beyond tobacco emissions, extending to inhalable or aerosolized substances, including pharmaceutical research.

#### (b) Complete dose–response profiling in a single batch

Miniaturization enables full dose–response curves from a single exposure experiment, significantly reducing the number of replicates required to determine toxicity thresholds or PoDs. However, preliminary range-finding remains essential to effectively exploit the aerosol dilution range.

#### (c) Minimizing background stress via controlled vehicle mass flow

Maintaining vehicle and test aerosol humidity is critical for physiological relevance, as dry exposure atmospheres induce non-specific epithelial stress that can skew sensitive biological endpoints. Adequate humidification is therefore essential not only for cytotoxicity assessments but also for reliable evaluation of mechanistic effects at sub-toxic exposure levels [40].

#### (d) Reducing operator variability through automation

Automated operation minimizes handling errors, enhances reproducibility, and facilitates process standardization via defined operating procedures. This is critical for inter-laboratory comparability and ultimately regulatory acceptance.

#### (e) Method consistency across aerosol types

The system successfully differentiated toxicity profiles of both combustible cigarette smoke (1R6F) and HTP-aerosol (IQOS), demonstrating in principle its applicability across aerosol classes, which is a pivotal step toward broader method standardization.

### Considerations for aerosol dosimetry and extended exposure

Accurately determining the administered dose remains a core challenge in ALI exposure. Inhalation is inherently an intermittent and dynamic process—particularly in smoking, where repeated inhalation episodes generate aerosol peak concentrations in the respiratory tract and the distal lung. Estimating the actual deposited dose at the epithelial level in vivo is technically complex and remains a major limitation [28, 41, 42].

To experimentally approximate real-world inhalation exposures and facilitate clear interpretation of observed effects, current ISO and CORESTA guidelines in principle support these to two dosimetry approaches:

- *Peak aerosol concentration*, representing the maximum aerosol level delivered during discrete injection events (e.g., 8 s of whole aerosol input into the vehicle airflow), and
- *Mean aerosol concentration*, averaged over the full exposure cycle (inhalation–pause–exhalation).

Each method reflects a compromise in approximating the biologically effective dose and yields different safety margins when used to derive PoDs. While mean concentration is often favored for defining threshold values, peak concentrations better capture transient, high-exposure episodes that may cause acute, irreversible effects. Thus, both perspectives should be considered to ensure a balanced hazard assessment.

Technologically, the capability to accurately simulate human-relevant exposure scenarios—particularly those intended for regulatory hazard evaluation—continues to advance. Especially during extended exposure durations under continuous-flow conditions (e.g., exceeding 1.5 h), system weaknesses may become evident, and minor inconsistencies such as thermal bridging or material irregularities can become more pronounced and noticeable.

In this context, the extended exposure conditions used for IQOS aerosol assessment—comprising 21 tobacco sticks and an equivalent exposure duration of 84 minutes—represent extreme settings, especially when applied to the sensitive alveolar A549 cell model. Despite this, only a very slight tendency toward background impact was observed (Fig. 4b). Additionally, more complex in vitro test systems representing the upper respiratory tract—more resilient due to mucin-mediated epithelial protection [43]—may be better suited for repeated or prolonged exposures. Such advanced 3D tissue models like MucilAir® are promising candidates for simulating “sub-chronic” or repeat-dose inhalation scenarios [6, 44,45]. It is especially the ongoing development of novel consumer products—often accompanied by limited toxicological data but potentially higher PoDs—that underscores the need for in vitro models capable of investigating subtle, low-dose effects [46,47].

However, while the sensitivity of analytical and bioassay techniques continues to advance, in vitro lung models—and particularly the exposure technologies they depend on—have yet to reach the same level of maturity. This disparity underscores the critical need for validated, high-performance exposure systems as a prerequisite before selecting more sophisticated and biologically relevant in vitro or ex vivo test systems.

### The proof-of-principle study provides hazard profiling of novel tobacco products (NTPs)

To assess the performance of the novel exposure system, a proof-of-principle study using the combination of a well-characterised reference (cigarette smoke, 1R6F) and a widely accepted epithelial cell model (A549) was conducted. This approach ensured a stable baseline by minimizing confounding variables unrelated to the system’s technical operation. The internal validation was then extended by comparing 1R6F emissions with those from a representative next-generation HTP, IQOS. While no fundamental new insights were expected regarding the established in vitro toxicity of cigarette smoke, the comparison demonstrated the HTES’s capability to characterize and differentiate aerosols with limited toxicological data. Moreover, using an extended bioassay panel under acute exposure conditions, the toxicity profile of IQOS revealed approximately 5% of the cytotoxic, genotoxic, and immunomodulatory effects observed for 1R6F (Table 2). Nevertheless, the presence of electrophilic compounds in the IQOS aerosol was confirmed—consistent with the heating process reaching temperatures up to 350 °C, sufficient to cause pyrolytic degradation of organic constituents. The formation of volatile toxic compounds, including reactive aldehydes (Fig. 3), appears to result from dehydration and oxidative transformation of humectants such as propylene glycol and glycerine [48]. These findings challenge the traditional paradigm of tobacco smoke carcinogenicity, which has primarily focused on polycyclic aromatic hydrocarbons (PAHs) and nitrosamines. Increasing evidence points to electrophilic aldehydes as major contributors to inhalation carcinogenicity [49], although their impact must be evaluated within the complex context of mixed-exposure toxicology..

Notably, some substances occur at higher concentrations in HTP aerosols than in combustible cigarette smoke, and others are uniquely present in HTPs [50, 51]. The toxicological relevance of these compounds—particularly in relation to long-term or cumulative exposure—remains poorly understood. This underscores the importance of establishing robust, transferable in vitro platforms to efficiently screen and prioritize emerging products for further toxicological evaluation.

While often marketed as safer alternatives to combustible tobacco cigarettes, our findings are consistent with existing evidence, including in vivo studies, demonstrating that HTPs like IQOS still pose potential health hazards. These include the potential to induce cytotoxicity, genotoxicity, and immunomodulatory effects [50, 52].

### Current methodological limitations and considerations for predictive pulmonary hazard assessment

The primary strength of in vitro methods lies in their ability to enable early identification of hazards or efficacy of airborne substances. However, these methods are not yet capable of fully replicating in vivo conditions or predicting real-world outcomes with complete accuracy. For instance, transitioning cells from submerged to ALI culture has been shown as the first step to induce physiological and morphological adaptations in lung cells or 3D models and can even drive terminal differentiation into functional, mucociliated airway epithelia [50, 53, 54]. Nevertheless, combining ALI culture systems with automated aerosol exposure under continuous-flow conditions already represents a critical advancement toward more physiologically relevant testing environments. Despite these advances, several limitations persist. The approach is technically demanding and not universally applicable, as many laboratories lack the infrastructure or expertise for aerosol-based exposure systems. Consequently, most preclinical studies on tobacco and related products still rely on conventional submerged culture techniques coupled with offline aerosol sampling. These traditional methods fundamentally diverge from the native inhalation process in multiple ways. Notably, the trapping and extraction of aerosols into liquid phases inherently alters their composition—favoring water-soluble fractions and excluding particulate components— thereby distorting the aerosol’s native physicochemical profile and bioavailability [50, 53–55].

A key advantage of advanced ALI exposure systems is their capability for precise control and comprehensive characterization of whole aerosol delivery. This allows a more accurate representation of the dynamic exposure kinetics observed during inhalation than is typically achievable in vivo [56]. To maximize the predictive power of such systems, it is essential to narrow the technical parameters of aerosol delivery. This includes mimicking human inhalation mechanics, ensuring proper conditioning of test aerosols (e.g., through humidification and mixing), alongside real-time dosimetry.

As NAMs gain traction in inhalation toxicology, the need for harmonized and standardized testing strategies becomes increasingly apparent. Such standardization is essential not only for reproducibility and data comparability in academic research but also for regulatory acceptance. However, their broader implementation hinges on the successful inter-laboratory transferability and procedural standardization. Ultimately, the decision to harmonize and adopt a particular exposure methodology for regulatory purposes lies with governing authorities. Academia plays a pivotal role in generating evidence-based data and formulating actionable recommendations that steer the development and implementation of predictive, human-relevant toxicological methods.

## Conclusion

The experimental data confirm that the technical design has achieved a high technological readiness level, enabling significant advancements in identifying the safety and health hazards of whole aerosols. The modular platform from VITROCELL Systems—particularly it’s innovative high-throughput exposure system—has demonstrated robust and reliable performance in delivering complex aerosols for effect-based in vitro studies. It probably represents the most advanced evolution of existing medium/high-throughput exposure systems based on continuous-flow ALI technology [13].

The platform is capable of detecting fine-scale toxicological responses and mechanistic effects, including DNA damage and immunomodulation. This pathway-based approach enhances hazard assessment and drug development by enabling the study of disruptions in critical cellular processes, as described by adverse outcome pathways (AOPs)—an important advancement in pharmacology and active ingredient screening.

Given the diversity of technologies, experimental setups, and bioassays currently available, this study displays the state-of-the-art in continuous-flow ALI exposure systems. The proof-of-concept not only validated the methodology but also provided meaningful insights into the potential health hazard associated with NTPs. The HTES platform unlocks new applications by effectively evaluating aerosols from diverse sources and categorizing specific stressors based on the data obtained. It enables high-content screening (HCS), broadening the range of endpoints and increasing predictive accuracy. In situ assays—such as γ-H2AX foci analysis—offer mechanistic insights that extend well beyond first-tier hazard assessments. While computational in silico models remain valuable for extrapolating in vitro data to human exposures, the HTES delivers a critical advantage: the ability to generate empirical dose–response data, driven by its high-throughput and automation capabilities.

Its performance, enabling rapid screening and—if required—mechanistic follow-up studies, addresses a critical gap in toxicological and pharmaceutical research by providing a reliable and versatile in vitro lung model for inhalation exposure studies [44].

Even though a primary objective was to evaluate the HTES, the experimental data obtained from the investigated test items provided valuable new insights into the growing diversity of tobacco products. This expanding product range complicates tobacco control efforts, as regulators, industry, and independent scientists often assess new products primarily by comparing their toxicity and health effects to those of combustible cigarettes, thereby emphasizing relative rather than absolute hazard. While IQOS is supported by extensive manufacturer data and some independent research, this study contributes to the weight of evidence by directly comparing HTPs to combustible cigarettes. The comparative analysis of tobacco products underscores the urgent need for harmonized testing protocols for NAMs to ensure consistent, reliable, and regulatory-compliant evaluation across product types. Inconsistencies in experimental methodologies continue to create discrepancies in the assessment of health hazards associated with new consumer products, thereby hindering the ability to draw definitive conclusions [52].

## Figures and Tables

**Table 1:** Technical specifications and exposure variables defining the actual dose and aerosol concentration levels (% vol) used in ALI exposure experiments with 1R6F cigarette smoke and IQOS aerosol

	Exposure duration (min)	Number of puffs		Dilution levels											
				Clean Air	11	10	9	8	7	6	5	4	3	2	1
1R6F	28	56	Mean conc	0.0	1.4	1.6	1.8	2.2	2.7	3.6	4.3	5.3	6.9	10.0	18.0
			Peakconc	0.0	3.4	3.9	4.6	5.5	6.8	9.0	10.7	13.3	17.3	25.1	45.2
IQOS	84	168	Mean conc	0.0	3.4	4.0	4.9	6.3	7.3	8.7	10.8	14.1	18.6	27.5	52.4
			Peak conc	0.0	5.3	6.2	7.6	9.7	11.3	13.4	16.5	21.6	28.6	42.2	80.5

Both the mean and peak aerosol volume percentages (% vol) are provided to facilitate comparison between test items.

**Table 2:** Toxicological evaluation matrix showing endpoint-specific benchmark doses (BMD) and their lower confidence limits (BMDL) derived from nonlinear regression using the Hill model

Toxicity endpoint (Assay)	Point of departure BMDL (BMD)		Response factor (% of 1R6F)*
	1R6F	IQOS	
Cytotoxicity (LDH)	1.47 (1.84)	8.37 (9.48)	5.85
Genotoxicity (Comet assay)	5.69 (5.76)	37.98 (40.16)	4.99
Immunomodulation (ELISA)	1.53 (1.71)	8.72 (9.10)	5.85

These values were selected as PoDs for each biological endpoint. Differential response factors were calculated by normalizing IQOS-derived BMDL values to those of 1R6F, which was set to 100%. For the immunomodulation endpoint, BMDL values were based on MCP-1 secretion, a representative marker of chemokine-mediated responses to aerosol exposure.

* The response factors were adjusted by a factor of 3 to account for the longer exposure duration in the IQOS experiments (see Sect. 3.4)

## Data Availability

The datasets used and/or analysed during the current study are available from the corresponding author on reasonable request.

## Abbreviations

ALI: Air–liquid interface
AOP: Adverse outcome pathway
BMD/BMDL: Benchmark dose/benchmark dose lower confidence limit
BSA: Bovine serum albumine
HTES: 96-Well high throughput exposure system
HTS: High troughput screening
HTP: Heated tobacco product
NAM: (Animal-free) new approach methodology
NTP: Novel tobacco product
PBS: Phosphate buffered saline
PFA: Paraformaldehyde
PoD: Point of departure
RT: Room temperature
TPM: Total particulate matter
